# Qinggan Huoxue Recipe attenuates Alcoholic Liver Disease by suppressing PI3K/AKT signaling pathway based on network pharmacology

**DOI:** 10.7150/ijms.80329

**Published:** 2023-01-31

**Authors:** Junmin Wang, Yifei Lu, Caiyun Zhang, Shuxia Tian, Hongjiao Xiang, Peilun Ding, Junming Chen, Guang Ji, Tao Wu

**Affiliations:** 1 Minhang Hospital, Fudan University, Shanghai 201199, China.; 2 Institute of Interdisciplinary Integrative Medicine Research, Shanghai University of Traditional Chinese Medicine, Shanghai 201203, China.; 3 Institute of Digestive Disease, Longhua Hospital, Shanghai University of Traditional Chinese Medicine, Shanghai 200032, China.

**Keywords:** qinggan huoxue recipe, alcoholic liver disease, network pharmacology, PTEN/PI3K/AKT, traditional Chinese medicine

## Abstract

Qinggan Huoxue Recipe (QGHXR) is originated from Xiao Chaihu Decotion. Many experimental studies have confirmed that QGHXR can significantly alleviate the symptoms of alcoholic liver disease (ALD), but the detailed mechanism is still unclear. Using traditional Chinese medicine network pharmacology analysis system database and animal experiments, we found that 180 potentially chemical compositions and 618 potential targets were screened from the prescription, which shared 133 signal pathways with ALD. Through animal experiments, it was found that QGHXR could reduce the liver total cholesterol (TC), serum TC, alanine aminotransferase, aspartate aminotransferase of ALD mice, reduce the lipid droplets and inflammatory injury of liver tissue. Meanwhile, it can also increase PTEN, decrease PI3K and AKT mRNA levels. In this study, we obtained the targets and pathways of QGHXR in the treatment of ALD, and preliminatively verified that QGHXR may improve ALD through PTEN/PI3K/AKT signaling pathway.

## Introduction

Alcohol consumption is a major global health burden and a key cause of the development of alcohol liver disease (ALD) 1. With the prevalence of alcohol culture, average alcohol consumption has increased year by year, leading to an increased risk of ALD 2. ALD includes a wide range of clinical symptoms, including alcoholic steatosis, hepatitis and fibrosis, and can progress to cirrhosis and even hepatocellular carcinoma 3. In the United States, recent studies have reported that about 40% of liver cirrhosis-related deaths can be attributed to ALD, and the 3-month mortality rate of severe alcoholic hepatitis is about 50%, suggesting that ALD can be fatal without active treatment and intervention 4, 5. Due to the great harm of ALD, many studies have explored the pathogenesis and development of ALD. The current academic point of view is that liver injury, inflammation and liver fibrosis are vital nodes in the progression of ALD 3. However, at present, the potential molecular mechanism of defense against alcohol-induced liver damage is still unclear, and there is no FDA-approved drug therapy for patients 6. Therefore, it is urgent to find new therapeutic targets.

A growing number of studies have shown that traditional Chinese medicine (TCM) is useful in the treatment of ALD. Several studies have reported the health-beneficial effects of dietary phytochemicals, namely polyphenols, to prevent various diseases 7.

For example, astragalus saponins and astragalus polysaccharides were found to reduce liver injury in mice with ALD 8. Kinsenoside reduced alcoholic liver injury by decreasing oxidative stress markers (3-Nitrotyrosine and 4-hydroxynonenal), inhibiting endoplasmic reticulum stress (ERS), and upregulated AMPK-dependent autophagy 9. Network pharmacology helps to find that Trikatu Churna can pass through a variety of signal pathways, including advanced glycation end products (AGE-RAGE), Hypoxia-inducible factors (HIF-1), Nuclear factor kappa-light-chain-enhancer of activated B cells (NF-Kappa B), and Phosphatidylinositol 3-kinase/protein kinase B (PI3K/Akt) signaling to exert its anti-inflammatory, antioxidant and anti-apoptotic role in treating ALD 10. Based on the investigation of the pathological factors of ALD patients, scholars summarized the TCM syndrome of different patients with ALD from the perspective of TCM, and put forward that “dampness-heat and blood stasis” is the basic pathogenesis mechanism of ALD, so as to establish “clearing liver and promoting blood circulation” as the basic treatment plan of ALD. According to the rich experience of Xiao Chaihu decoction in the treatment of ALD, the study group developed a practical prescription for prevention and treatment of ALD, Qinggan Huoxue Recipe (QGHXR), in Longhua Hospital of Shanghai University of traditional Chinese Medicine. According to the basic theory of traditional Chinese medicine, *Bupleurum abchasicum Manden* and *Scutellaria baicalensis Georgi* can clear liver and remove dampness, and are used as the main drugs. The second main drugs are *Salvia miltiorrhiza Bge* and *Trionyx sinensis Wiegmann*, which can activate blood circulation and remove blood stasis. As an auxiliary drug, *Pueraria lobate (Willd.) Ohwi* can relieve muscle soreness, thirst and promote the circulation of Yang Qi. The prescription is used for clearing heat and removing dampness, promoting blood circulation and removing blood stasis 11.

Many experimental studies have confirmed that QGHXR can significantly alleviate the symptoms of ALD. Clinical randomised controlled trial study confirmed that it can significantly improve the symptoms and signs of anorexia, nausea, vomiting and jaundice in patients with ALD 11. Previous study found that QGHXR could antagonize the damage of ethanol to liver by accelerating the metabolism of ethanol in the liver and alleviating lipid metabolism disorders and preventing the accumulation of lipids in the liver 11-13. In addition, experimental studies have shown that QGHXR can alleviate ALD by inhibiting lipopolysaccharide-Kupffer cell signaling, and it also could inhibit epithelial-mesenchymal transition (EMT) by inhibiting TGF-β1/Smad signaling pathway to alleviate alcoholic liver fibrosis 14, 15. Further research found that the active ingredients baicalin and puerarin from QGHXR can inhibit EMT via the TGF-β1/Smad3 pathway *in vitro* to improve alcoholic liver fibrosis 16. Lu et al. recently found that QGHXR can improve ERS by regulating the expression level of liver X receptor (LXR) and lysophosphatidylcholine acyltransferase 3 (LPCAT3), thus reducing alcohol-induced hepatic steatosis inflammation and liver injury 17. However, due to the large number of drugs and complex chemical components of QGHXR, the detailed mechanism of QGHXR on ALD is not completely clear. Network pharmacology is an approach which establishes a network by setting up signal nodes, and further analyzes the network to explain the disease process and the effect of drugs on the body, so as to study the mechanism of drug action. In recent years, network pharmacology plays an important role in the research of TCM, such as screening the active components, predicting the targets, expounding the mechanism of action of prescription and so on.

**Figure 1:**
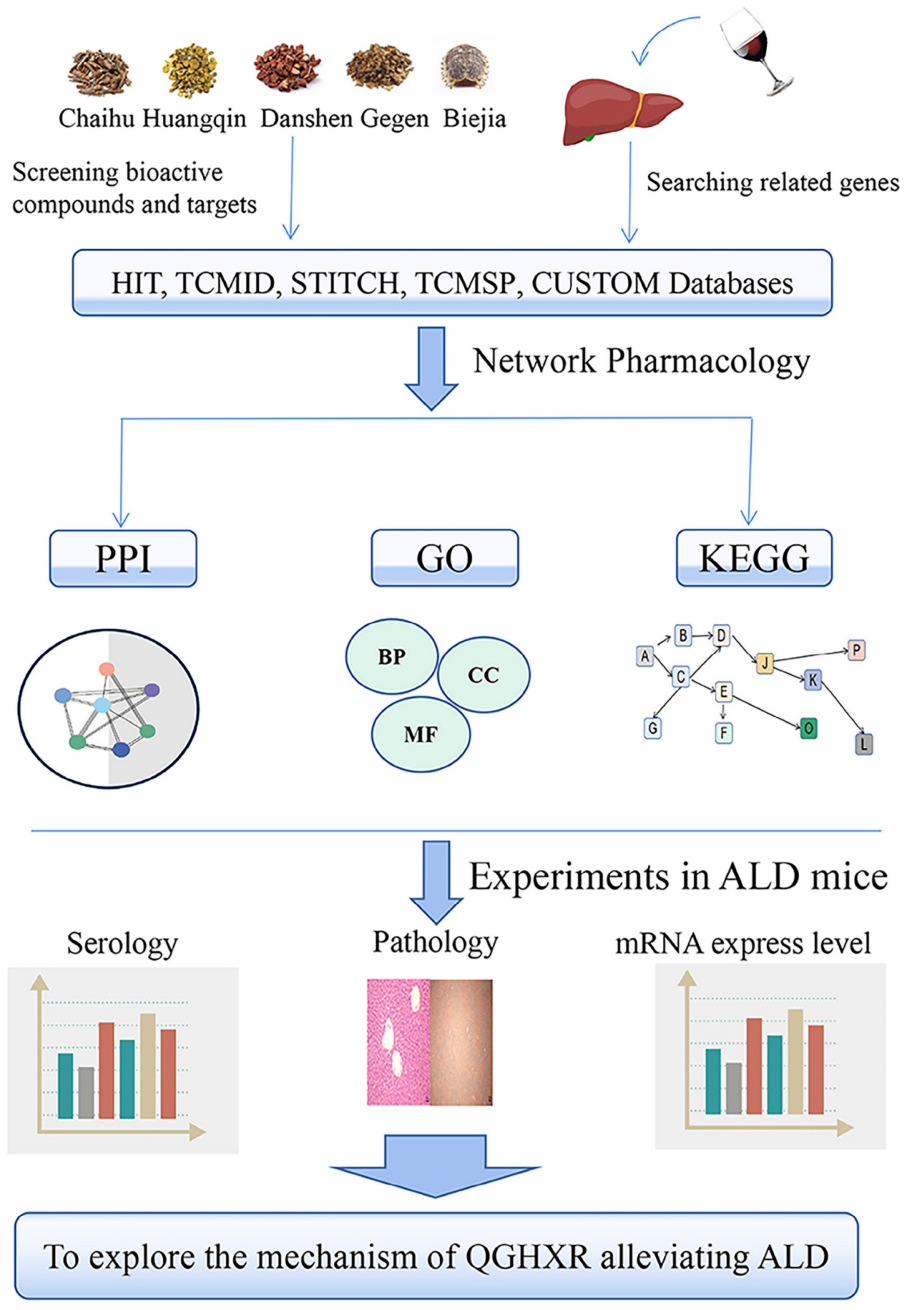
The detailed flow chart of the study.

In previous studies, we have found that QGHXR can improve ALD and liver fibrosis through LXR-LPCAT3-ERS and TGF-β1/Smad signaling pathway, respectively, but all the mechanisms have not been explored clearly. In the present study, we first collect the active components of QGHXR using network pharmacology, and then collect the gene targets of these active components by the database TCM Network Pharmacology Analysis System (TCMNPAS). At the same time, the related gene targets of ALD were enriched, and then the common genes of both were enriched by KEGG pathway from the database. Finally, the pathway was verified by animal experiments *in vivo*. It is the first time to collect the active components and gene targets of QGHXR and use network pharmacology to explore the mechanism of QGHXR improving ALD. It was found that 180 potentially chemical compositions and 618 potential gene targets were screened from the QGHXR and the all genes shared 133 signal pathways with ALD. Through animal experiments, we found that QGHXR could reduce the liver total cholesterol (TC), serum TC, alanine aminotransferase (ALT), aspartate aminotransferase (AST) of ALD mice, reduce the lipid droplets and inflammatory injury of liver tissue and we preliminarily verified that QGHXR may improve ALD through PTEN/PI3K/AKT signaling pathway. The detailed flow chart of the study is shown in **Figure 1.**

## Materials and Methods

### Network Pharmacology

#### Collected chemical components and target genes of QGHXR

TCMNPAS, a network pharmacological analysis system of TCM, was independently developed and designed by Professor Yang Ming in Longhua Hospital, Shanghai University of Traditional Chinese Medicine (TCM Network Pharmacology Analysis System v1.0 [CP/CD], Copyright Registration No., 2019SR1127090, http://54.223.75.62:3838/). It is mainly used for network pharmacological analysis of TCM compound prescription and its chemical ingredients, and provides one-stop solution for complex data analysis. The five herbs of QGHXR,* Bupleurum abchasicum Manden., Scutellaria baicalensis Georgi, Salvia miltiorrhiza Bge, Pueraria lobate (Willd.) Ohwi,* and* Trionyx sinensis Wiegmann* were input into the retrieval module and HIT, TCMID, STITCH, TCMSP and CUSTOM databases were selected. The QED value was set to 0.2, and the compound-target association score was set to 400. (Limitations: the database does not include the chemical composition of animal drugs. Most of traditional Chinese medicines come from plant leaves, stems, roots, etc., but a few medicines are fur or tissue from animals, called animal drugs. The *Trionyx sinensis Wiegmann* comes from the shell of a turtle, thus there is no research data in the database. It is one of the limitations in the present study).

#### Collected related targets of ALD

The names (alcoholic liver injury, alcoholic liver disease, middle alcoholic injury, alcoholic fatty liver, alcohol hepatitis, alcoholic hepatic fibrosis, alcoholic cirrhosis) of the disease related to ALD are inputted into the disease ID retrieval module of TCMNPAS, which automatically connects with the GeneCard database (https://www.genecards.org/) to obtain the disease ID, and then inputs the obtained ID into the disease gene retrieval module of TCMNPAS to automatically obtain the related gene targets of ALD.

#### Constructed drugs-chemical components-gene targets-disease network

The chemical components and potential targets of QGHXR obtained from TCMNPAS database, as well as the information of ALD targets were sorted out, and QGHXR-chemical components-targets-ALD network map was established by Cytoscape 3.8.2.

#### Constructed protein-protein interaction (PPI) network

The potential targets of QGHXR and ALD targets information obtained from TCMNPAS database were sorted out and imported into STRING (https://string-db.org/) to construct PPI network.

#### Enrichment analysis of KEGG signaling pathway and GO biological process

The gene targets of QGHXR and ALD related gene targets obtained from TCMNPAS database were imported into the GO and KEGG database modules respectively, and the hypergeometric distribution model *P*-value was used to evaluate whether GeneSet was significantly associated with gene ontology. False discovery rate (FDR) adjusted *P* value (*P.* adjust) was used to reflect the association strength between targets and gene ontology, and *P*. adjust <0.05 was considered as a significant association, and GO enrichment and KEGG signaling pathway were obtained.

### Experimental verification of QGHXR attenuating ALD

#### Animals

Thirty male C57BL/6 mice, SPF grade, 8 weeks old, were provided by Shanghai SLAC Laboratory Animal Co., Ltd, and reared in the Animal Experiment Center of Shanghai University of TCM (Animal Qualification Certificate No: 20170005030600). All animal experiments were approved by the Ethics Committee of Shanghai University of TCM (Ethics No: PZSHUTCM200110003). Feeding environment of mice is as below: indoor standard clean, standard temperature 22 ± 2 °C, humidity 55% ~ 65%, light/dark cycle 12h/12h, ventilation cage regular autoclave, regular replacement of pad material.

#### Drugs and Reagents

QGHXR consists of Bupleurum abchasicum Manden., Scutellaria baicalensis Georgi, Salvia miltiorrhiza Bge, Trionyx sinensis Wiegmann and Pueraria lobate (Willd.) Ohwi (Jiangyin Tianjiang Pharmaceutical Co., Ltd., No. 19102524, No. 19070598, No. 19101349, No. 19090910, No. 19091622). QGHXR were concentrated and dissolved to 7.41 g/kg by using double distilled water. In our previous study, the dose has been explored and verified through experiments 17. Based on the previous studies, the contents of puerarin, baicalin, baicalein and wogonin were treated by HPLC (high-performance liquid chromatography) to ensure the quality control of Chinese herbal medicine. Lieber-DeCarli control liquid feed (F1259SP) and alcoholic liquid feed (F1258SP) were purchased from Bio-serv, USA company. 4% paraformaldehyde fixative was obtained from Beyotime biotechnology, Shanghai, China. Trichloromethane (10006818), isopropyl alcohol (80109218) and anhydrous ethanol (10009257) were purchased from Sinopharm Chemical Reagent Co., Ltd. Total RNA Extraction Kits (19211ES60) and Fluorescent dye (1120E03) come from Yeasen Biotech Co., Ltd. Reverse Transcription Kits (KR118) were purchased from TIANGEN BIOTECH (BEIJING) Co., LTD.

#### Group and Administration

There were 10 mice in each group (pair-fed, EtOH-fed, EtOH-fed+QGHXR). According to the mouse model of chronic and binge ethanol feeding (the NIAAA model) reference 18, each group was given Lieber-DeCarli control liquid diet (F1259SP, Bio-serv Company) for 5 days, then EtOH-fed+QGHXR and EtOH-fed group were fed with liquid diet containing 5% alcohol (F1258SP, Bio-serv Company) for 10 days, and the pair-fed group was given liquid control diet of the same volume. The EtOH-fed+QGHXR group were given intragastric administration of QGHXR with a dose 7.41g/kg once a day on the 6th-15th day, and the mice in the other groups were fed with the same volume of normal saline. According to the NIAAA model, we prepared a 31.5% (vol/vol) ethanol solution (0.25 g/ ml ethanol) by mixing 6.6 ml of 95% ethanol with 13.4 ml of water and a 45% (wt/vol) maltose dextrin solution by dissolving 9 g of maltose dextrin in a final volume of 20 ml of water. 1 g ethanol = 7 kcal, 1 g maltose dextrin (Bio-Serv) = 3.89 kcal. At 7-9 a.m. on the 16th day, the pair-fed group was given 45% maltose dextrin with a dose of 9g/kg, while the EtOH-fed group and EtOH-fed+QGHXR were given 31.5% alcohol with a dose of 5g/kg (For mice of the same weight, these two doses provide the same energy). After 9 hours, 1% pentobarbital sodium was injected intraperitoneally under anesthesia (50 mg/kg).

#### Detection of serum liver function and lipids

Mice were given 1% pentobarbital intraperitoneally and blood was collected by removing the eyes. The whole blood was kept at 4 °C for 3 hours for 2775 g/min, 15 min, and the supernatant was used to detect serum TG, TC, ALT and AST by Japanese Toshiba TBA-40FR automatic biochemical analyzer.

#### Detection of liver lipids and analysis of liver histomorphology

After animal blood collection, the mice were sacrificed by cervical isolation. The fresh liver was removed and then stored in the -80 °C refrigerator. 50 mg of liver tissue from each liver tissue sample of the three groups were cut into a 1.5 ml centrifuge tube, and then adding 2-3 magnetic beads and 500 μl of tissue homogenate (ethanol: acetone =1:1) were added. Next, 1.5 ml centrifuge tubes were placed in an ultrasonic homogenizer at 60 Hz, 60s, and then 3 times, in 4 °C refrigerators overnight. The next day, after centrifugation at 2775g/min for 15 min, 150 μl of supernatant was used for assays. The supernatant was detected by Toshiba TBA-40FR automatic biochemical analyzer. Two pieces of 1cm * 1cm liver tissue were cut and fixed in 4% paraformaldehyde, dehydrated and embedded into paraffin sections for hematoxylin-eosin staining (HE). Frozen liver sections were stained with oil red. The images were collected by an optical microscope (HORIBABX41, Japan).

#### Quantitative real-time PCR

According to the RNA extraction kits, the total RNA was extracted from 50mg liver tissue of each sample (n=6, six liver tissue samples were selected from each group, and the PCR results were performed 2 times.), and the total mRNA was reverse transcribed by one-step reverse transcription kit to synthesize cDNA. The expression level of each target gene was detected by fluorescence quantitative PCR amplification with SYBR Green Master Mix. The primer sequence is mPTEN: Forward Primer 5'-AAGACCATAACCCACCACAGC-3', Reverse Primer 5'-CCAGTCCGTCCCTTTCCAG-3', 124 bp; mPI3K: Forward Primer 5'-AAGCCATTGAGAAGAAAGGACTG-3', Reverse Primer 5'-ATTTGGTAAGTCGGCGAGATAG-3', 176 bp; mAKT: Forward Primer 5'-TGTCTGCCCTGGACTACTTGC-3', Forward Primer 5'-GGCGTTCCGCAGAATGTC-3', 166 bp; m β-actin: Forward Primer 5'-GAGACCTTCAACACCCCAGC-3', Reverse Primer 5'-ATGTCACGCACGATTTCCC-3', 263bp. The reaction conditions are as follows: 95 °C predenatured for 5 min, cycling once; 95 °C denatured for 10s, 60 °C annealed for 20s, 72 °C extended for 20s, and 40 cycles.

### Statistical methods

All data are expressed as mean ± SEM. GraphPad Prism 6 was used for statistical analysis. Statistical comparisons of multiple groups were performed by one-way analysis of variance (ANOVA). Comparisons between two groups were performed using two-tailed unpaired Student's *t* test. The *p* < 0.05 was considered statistically significant.

## Results

### Network Pharmacology

#### Predict chemical components and potential targets of QGHXR

According to the results of TCMNPAS database, there were 101 active compounds in Bupleurum abchasicum Manden., 55 active compounds in Scutellaria baicalensis Georgi, 47 active compounds in Salvia miltiorrhiza Bge and 9 active compounds in Pueraria lobate (Willd.) Ohwi. But Trionyx sinensis Wiegmannis was not included in the database. Different Chinese herbs had common gene targets. In order to count how many kinds gene targets of the four Chinese herbs, the same gene targets were counted once. The results showed that there were 180 active compounds and 29 active compounds overlapped in different drugs. A total of 618 genes were obtained in QGHXR after the repetition was removed (**Supplementary Table S1**).

#### Construct drugs-chemical components-potential targets and PPI network

**Figure 2:**
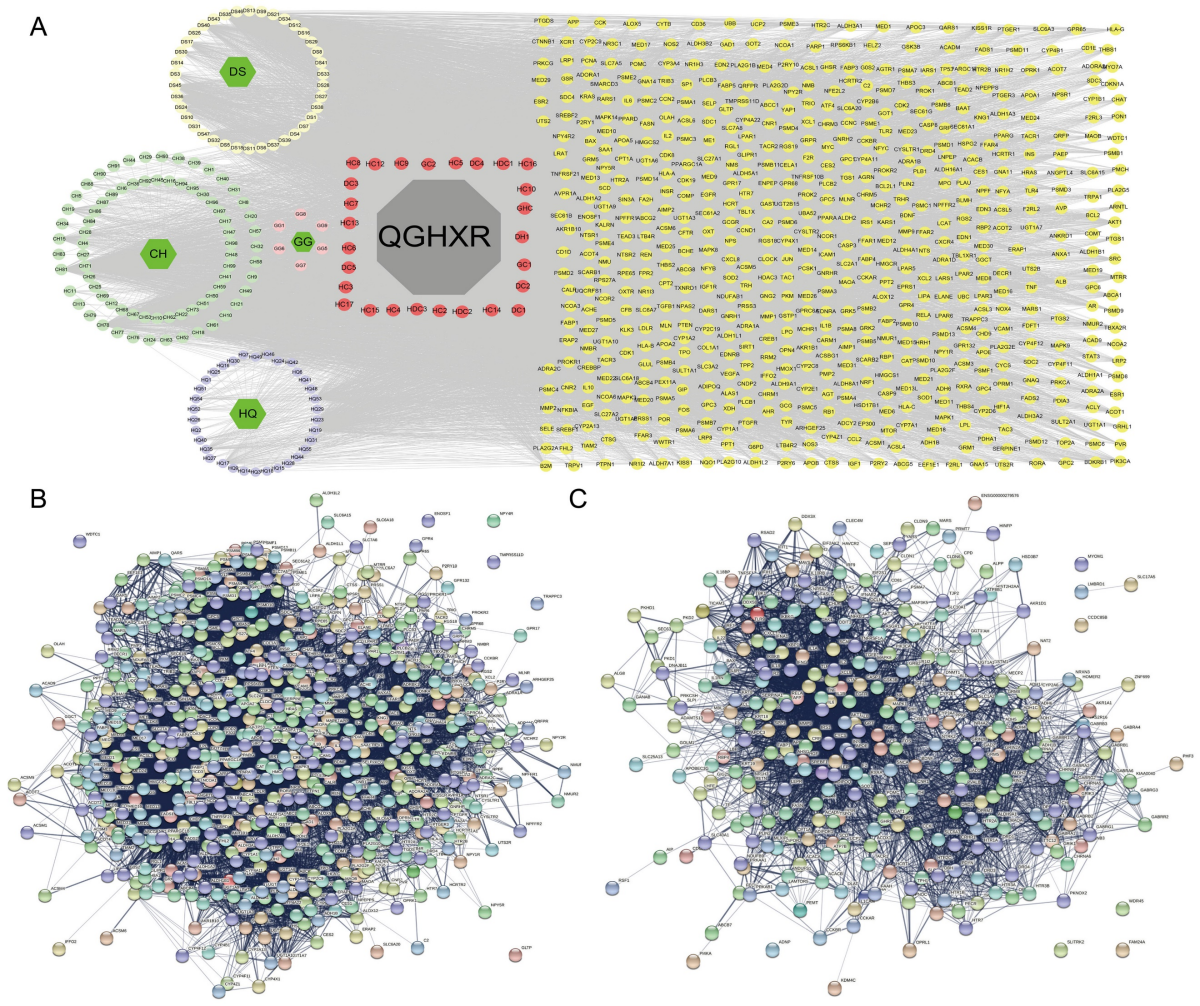
** Network pharmacological analysis of QGHXR and ALD. (A)** The drugs-chemical components-potential targets network. Gray shapes represent the prescription; green shapes represent the name of 4 drugs; purple blue shapes represent the chemical composition of HQ; light green shapes represent the chemical composition of CH; light yellow shapes represent the chemical composition of DS; pink shapes represent the chemical composition of pueraria; red shapes represent the common chemical composition; deep yellow represents all gene targets of QGHXR. **(B)** The PPI network of QGHXR. **(C)** The PPI network of ALD. Each circle represents each individual protein. Abbrevation: QGHXR, qinggan huoxue recipe; CH, chaihu (Bupleurum abchasicum Manden.); DS, danshen (Salvia miltiorrhiza Bge); HQ, huangqin (Scutellaria baicalensis Georgi); GG, gegeng (Pueraria lobate (Willd.) Ohwi).

The results of 180 chemical components and 618 potential action targets obtained from QGHXR were imported into Cytoscape 3.8.2 software and STRING database to construct drugs-chemical components-potential targets network **(Figure 2A)** and PPI network** (Figure 2B, Supplementary Table S1-S2).**

#### Construct PPI network of ALD

According to the TCMNPAS database, a total of 433 ALD targets were screened, and 316 disease targets were obtained after de-recombination. A PPI network was built through importing the results into the STRING database, and it is shown in **Figure 2C, Supplementary Table S3.**

#### PPI network between QGHXR and ALD targets

**Figure 3:**
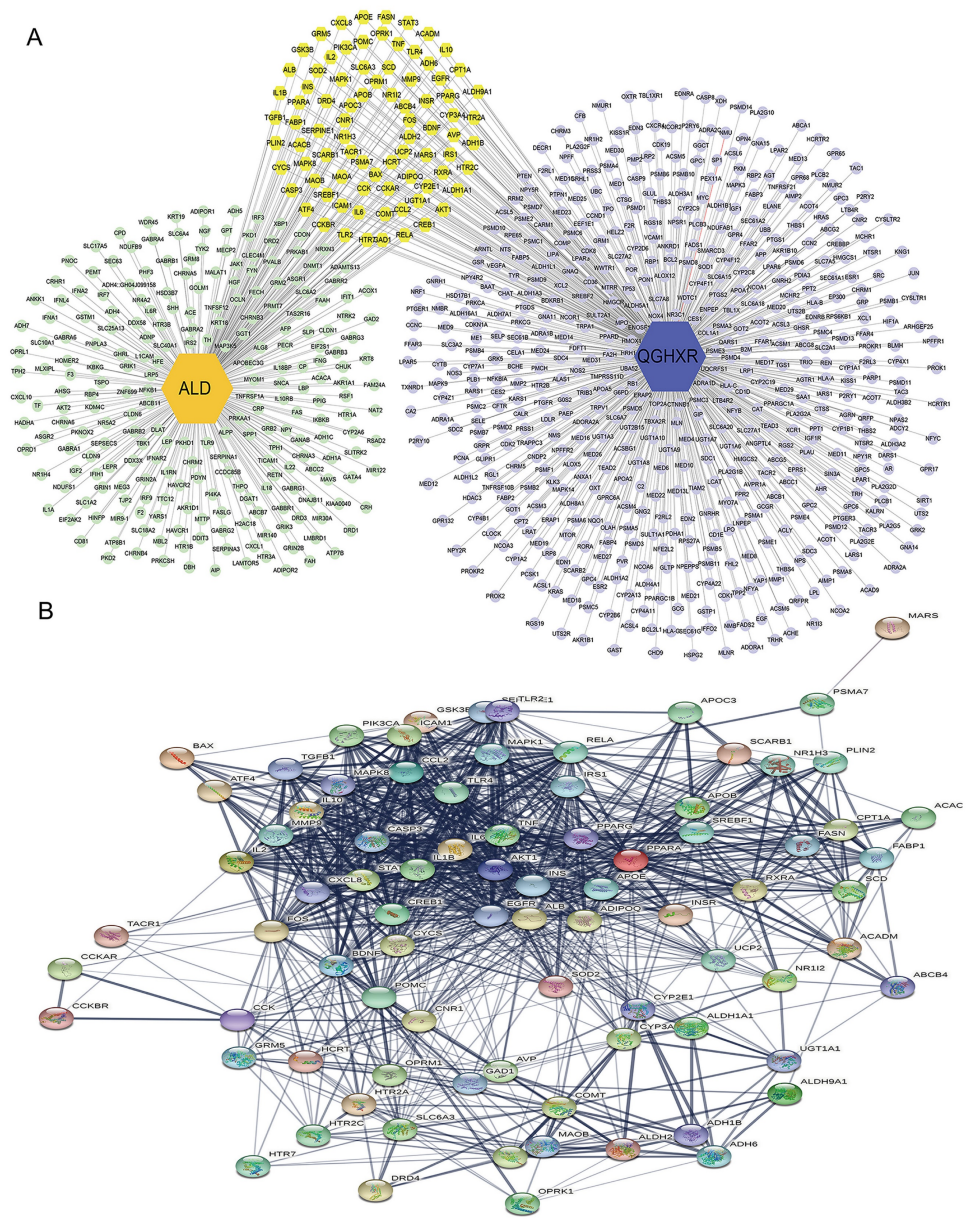
** Gene targets for both QGHXR and ALD. (A)** The QGHXR-targets-ALD network. Light green patterns represent gene targets of ALD; light purple patterns represent gene targets of QGHXR; and yellow patterns represent shared gene targets of both. **(B)** The PPI network of common targets in ALD and QGHXR. Each circle represents each individual protein.

83 gene targets shared by QGHXR and ALD were screened by Cytoscape **(Figure 3A, Supplementary Table S4)**, and then introduced into STRING database to establish the PPI for common components-disease targets **(Figure 3B)**.

#### GO function analysis and KEGG signal pathway enrichment analysis

**Figure 4:**
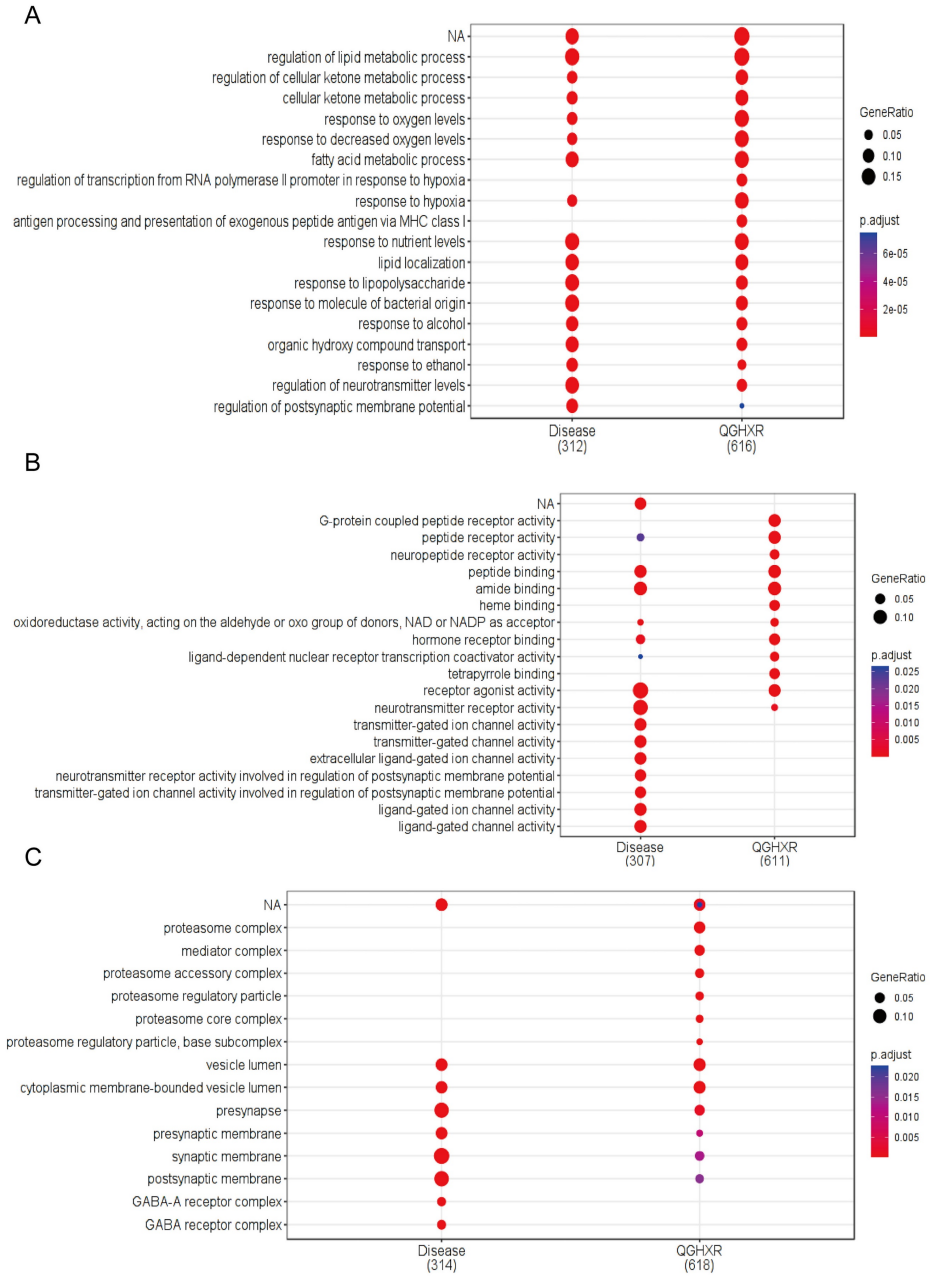
** GO enrichment analysis of shared gene targets for QGHXR and ALD. (A)** The GO pathways of QGHXR and ALD based on biological processes. **(B)** The GO pathways of QGHXR and ALD based on molecular functions. **(C)** The GO pathways of QGHXR and ALD based on cellular components.

GO enrichment analysis and KEGG pathway analysis were performed for relevant gene targets of QGHXR and ALD obtained from TCMNPAS database, with p <0.05. A total of 2592 compound GO pathways and 1814 disease GO pathways were obtained based on biological processes (BPs) **(Figure 4A, Supplementary Table S5)**, based on molecular functions (MFs) 238 compound GO pathways and 170 disease GO pathways** (Figure 4B, Supplementary Table S6)**, and based on cellular components (CCs) 112 compound GO pathways and 93 disease GO pathways** (Figure 4C, Supplementary Table S7).** Comprehensive GO enrichment analysis showed that the potential targets of QGHXR and disease targets showed co-correlation at the GO level, and the GO similarity between QGHXR and ALD genes was 74.23%.

**Figure 5:**
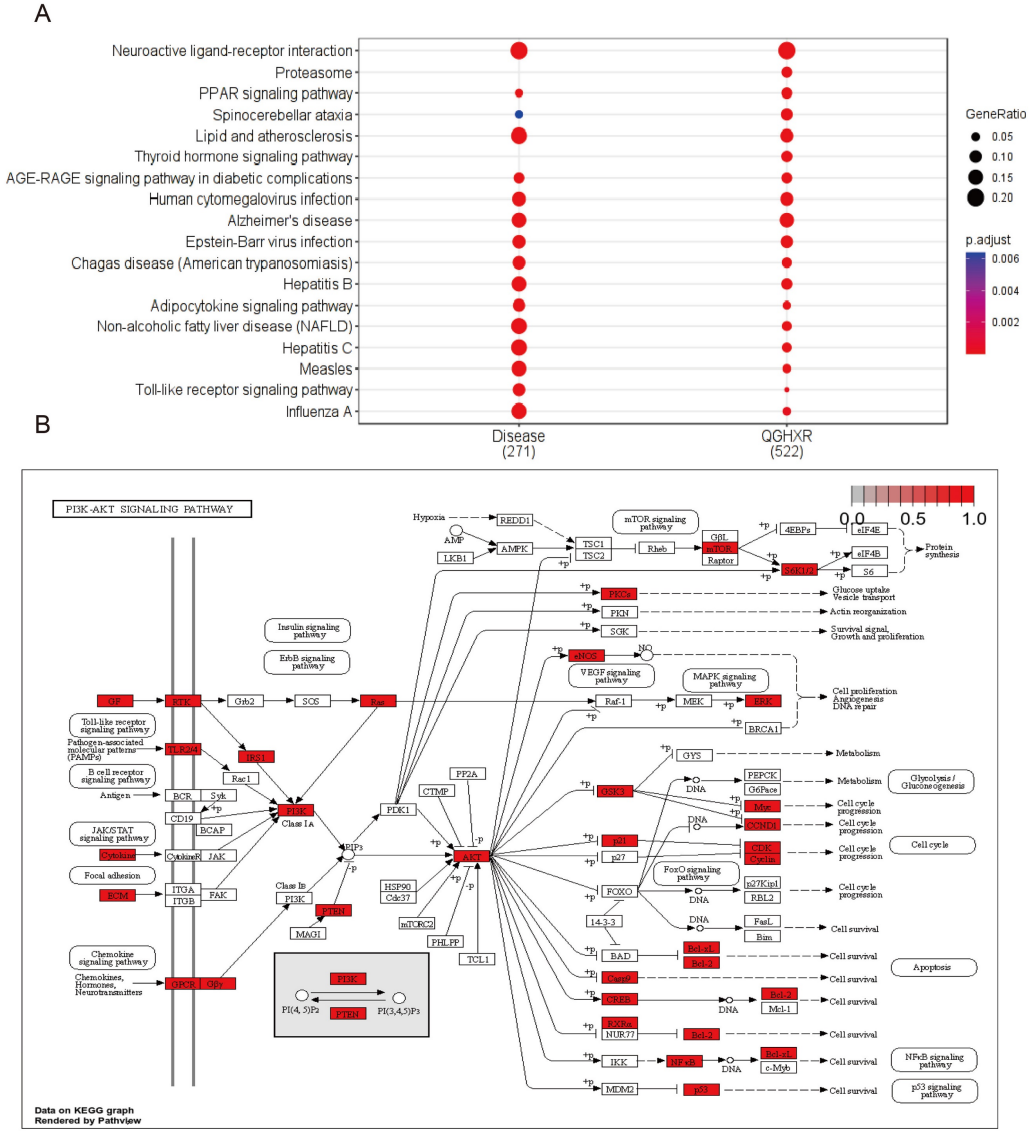
** Pathway enrichment analysis of common targets between QGHXR and ALD. (A)** The KEGG pathways of QGHXR and ALD. **(B)** The PI3K/AKT signal pathway.

KEGG pathway analysis showed that there were 133 common signal pathways between QGHXR and ALD **(Figure 5A, Supplementary Table S8-S9)**. The top ten common signal pathways were obtained according to GeneRatio **(Table 1)**, and 4 related signal pathways were screened. These were Neuroactive ligand-receptor interaction, Lipid and atherosclerosis, Alzheimer's disease, and PI3K/AKT signaling pathway **(Figure 5B)**. The similarity of KEGG signal pathway associated with QGHXR and ALD was 89.1%.

### Experiment

#### Effect of QGHXR on serum indications and liver lipids in ALD mice

**Figure 6:**
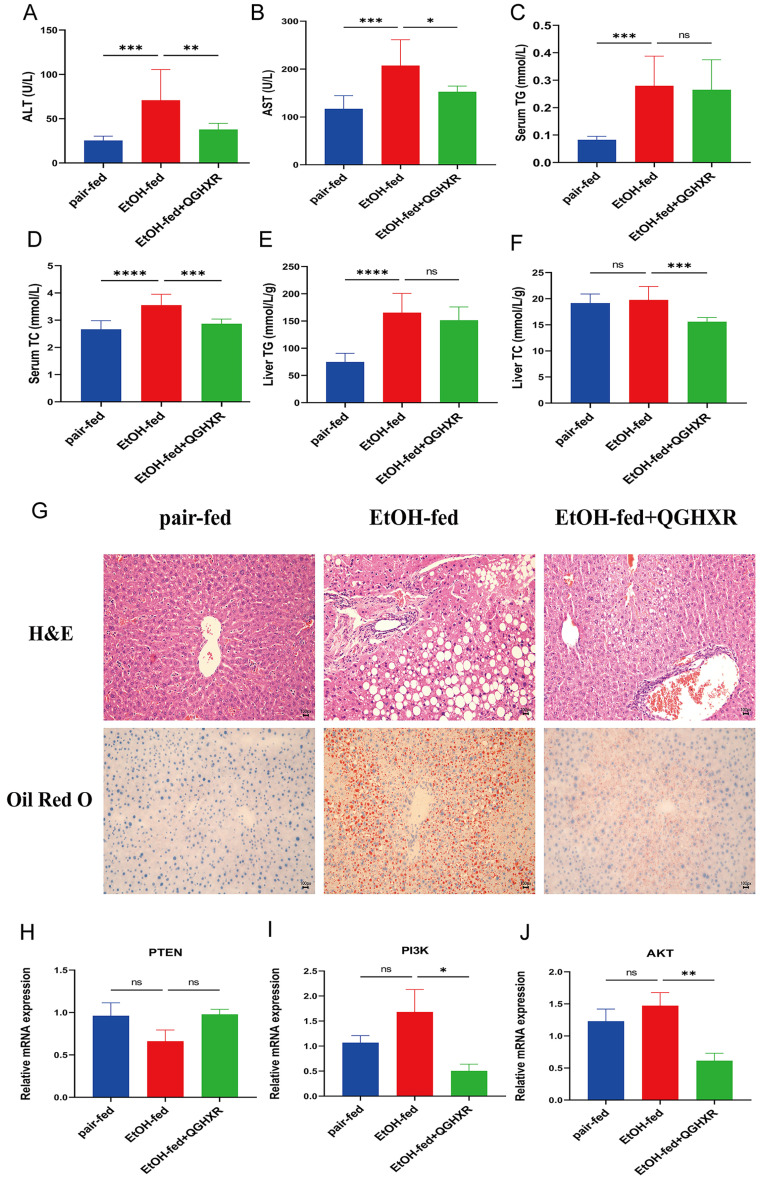
** QGHXR alleviates the development of ALD. (A)** Serum alanine aminotransferase (ALT) levels.** (B)** Serum aspartate aminotransferase (AST) levels.** (C)** Serum total triglyceride (TG) levels. **(D)** Serum total cholesterol (TC) levels. **(E)** Liver TG levels. **(F)** Liver TC levels. **(G)** The representative H&E and oil red O staining pictures in pair-fed, EtOH-fed and EtOH-fed+QGHXR groups (x400). **(H)** The mRNA expression of PTEN. **(I)** The mRNA expression of PI3K. **(J)** The mRNA expression of AKT. Compared with EtOH-fed group, *p<0.05, **p<0.01; ns, no significance.

It was shown that serum ALT, AST, TG, TC, liver TG levels were significantly increased in EtOH-fed group mice compared with pair-fed group mice. QGHXR significantly reduced serum ALT, AST, TC and liver TC in the EtOH-fed group mice, but had no significant effect on serum TG and liver TG levels** (Figure 6A-F).**

#### Effects of QGHXR on pathological changes of ALD mice

HE staining showed that compared with pair-fed group, steatosis with large vesicular fat droplets and hepatocyte necrosis appeared in EtOH-fed group, and more inflammatory cell infiltration could be seen in hepatic sinusoid, while EtOH-fed+QGHXR group had obvious improvement effect. Oil red O staining showed that there were much lipid droplets in the liver tissue of EtOH-fed group mice with hepatocyte expansion, indicating the existence of liver injury. Compared with EtOH-fed group, the pathological morphology of liver tissue in EtOH-fed+QGHXR group was obviously improved** (Figure 6G)**.

#### Effect of QGHXR on PTEN, PI3K, AKT mRNA expression in liver tissue of ALD mice

Compared with the pair-fed group, the PTEN mRNA expression had a downward trend in EtOH-fed group and the mRNA level of PI3K and AKT had an increasing trend, but there was no significant statistical significance. Compared with EtOH-fed group, the level of PTEN mRNA was higher in EtOH-fed+QGHXR group, while the mRNA of PI3K and AKT obviously decreased, but PTEN did not show statistical significance **(Figure 6H-J).**

## Discussion

Excessive and chronic alcohol intake can lead to many problems that affect a variety of physiological systems, including the immune, nervous, cardiovascular and digestive system 19, 20. Among many medical diseases related to alcohol consumption, ALD is the main cause of death 21. As there is no effective treatment for ALD, it is necessary to better understand the pathogenesis of ALD and provide effective therapeutic targets for the treatment of ALD.

QGHXR is composed of five traditional Chinese drugs, among which saikosaponin D, the main active ingredient of *Bupleurum abchasicum Manden.*, can protect the liver in alcoholic hepatitis mice with anti-inflammatory and anti-oxidation effects 22. Baicalin, the main active component of *Scutellaria baicalensis Georgi*, alleviates alcoholic liver injury through inhibiting oxidative stress, inflammatory response and regulating sonic hedgehog pathways 23. Salvianolic acid, the main active ingredient of *Salvia miltiorrhiza Bge*, can inhibit oxidative stress and inflammatory response, and alleviate liver fibrosis mainly by regulating Nrf2/HO-1 and NF-κB/IκBα signaling pathways 24, 25. Glaxin, the active ingredient of *Pueraria lobate (Willd.) Ohwi*, can improve liver glucose and lipid metabolism by inhibiting oxidative stress and inflammation and activating AMPK signaling 26, 27. In previous studies, it was shown that QGHXR could antagonize the damage of ethanol to liver by accelerating the metabolism of ethanol in the liver, alleviating lipid metabolism disorders and preventing the accumulation of lipids in the liver 11-13. We previously also found that QGHXR can alleviate ALD by inhibiting lipopolysaccharide-Kupffer cell signaling, and it also could inhibit EMT by inhibiting TGF-β/Smad signaling pathway to alleviate alcoholic liver fibrosis 14, 15. In addition, Lu et al. recently found that QGHXR can suppress ERS and improve ALD through LXR-LPCAT3 signal pathway 17.

**Figure 7:**
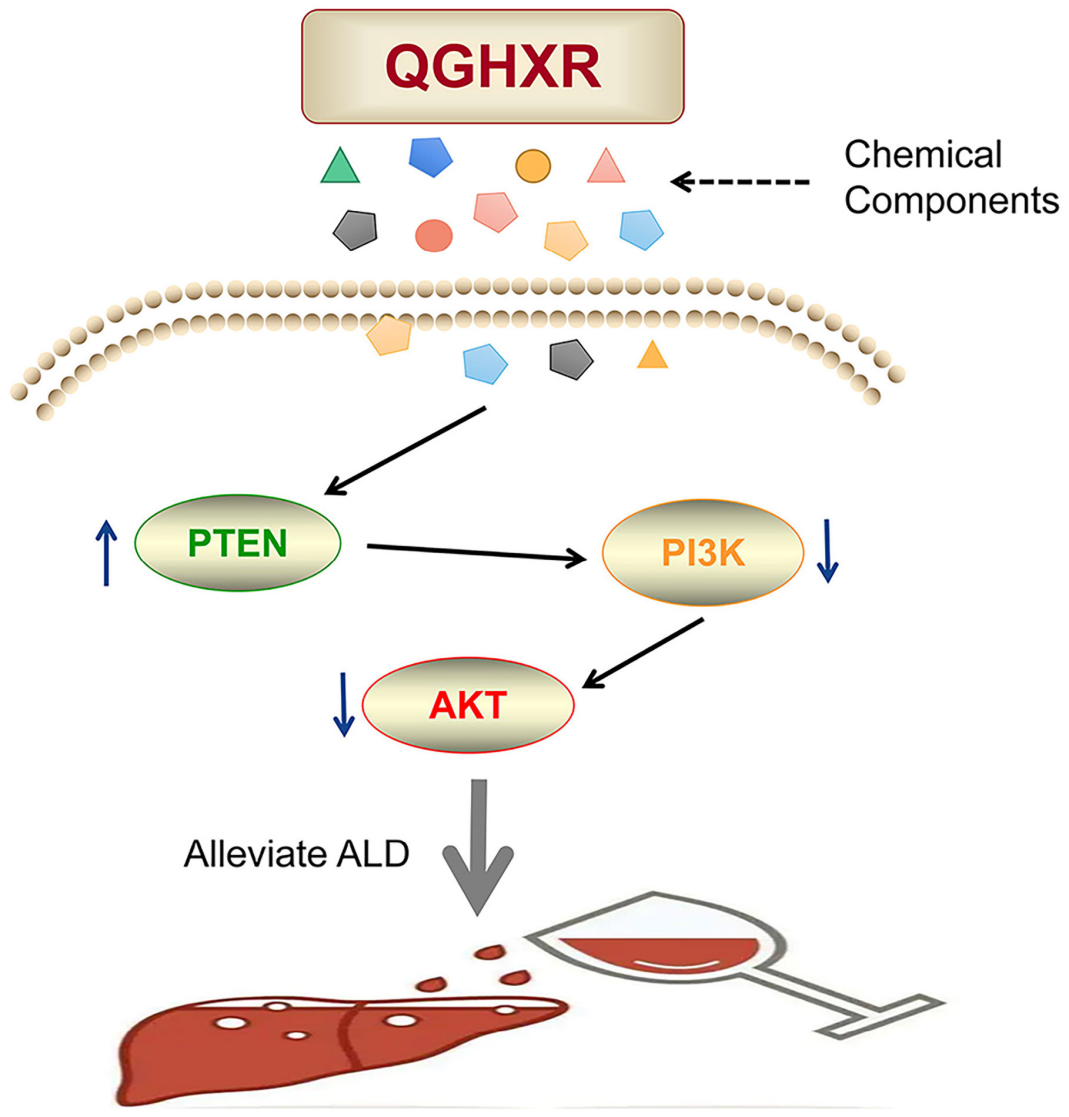
The diagram of pathological mechanism of QGHXR in improving ALD.

In this study, the mechanism of action between QGHXR and ALD was explored by network pharmacology. A total of 180 active components and 618 potential targets of QGHXR and 316 potential targets of ALD were obtained by using TCMNPAS database. Network maps and PPI maps of drugs-active components-targets-disease were constructed using STRING, Cytoscape and other tools. In addition, QGHXR and ALD were enriched by GO and KEGG signal pathways, and the results were based on BP 2592 compound GO pathway and 1814 disease GO pathways, MF 238 compound GO pathways and disease GO pathways, CC 112 compound GO pathways and 93 disease GO pathways. KEGG pathway analysis showed that there were 133 common signal pathways between QGHXR and disease, and the top 10 common signal pathways were obtained according to Gene Ratio, and 4 related signal pathways were screened. They are neuroactive ligand-receptor interaction, lipid and atherosclerosis, Alzheimer's disease and PI3K/AKT signaling pathway. Then, the PI3K/AKT signal pathway was verified in ALD mice. The results indicated that QGHXR could obviously reduce serum ALT, AST, TC and liver TG level, but had no obvious influence in serum TG and liver TC level. At the same time, it can reduce liver lipid droplets, inflammation and hepatocyte injury in ALD mice. More importantly, compared with EtOH-fed group, the mRNA level of PTEN was higher, while the mRNA level of PI3K and AKT apparently decreased in EtOH-fed+QGHXR group **(Figure 7)**.

However, there are some limitations in our present study. First of all, because the database did not collect the research data of Trionyx sinensis Wiegmann, we failed to enrich the active components and gene targets of Trionyx sinensis Wiegmann. Secondly, we did not further verify this pathway at the protein expression level and *in vitro* cell experiments, and it is better to further verify other signal pathways. Finally, we are temporarily unable to select the active components of QGHXR and carry out efficacy verification and mechanism exploration from *in vivo* or *in vitro* experiments.

## Conclusion and future perspectives

To sum up, this study revealed the mechanism of QGHXR improving ALD through multi-pathway and multi-target way, and proved that QGHXR can improve ALD through animal experiments *in vivo*, which may be carried out through PI3K/AKT signal pathway, which provides a research basis for subsequent work. In the future research, we will focus on the following aspects. First, we will continue to verify the protein expression of PI3K/AKT pathway. Second, *in vitro* cell experiments will be performed in order to make the research more scientific and rigorous. Third, it would be continued to further explore other signal pathways of QGHXR and ALD. Finally, it is suggested to further analyze the active components or mnonomers of QGHXR and explore their efficacy and mechanism. It is hoped that through more research, we can understand the clear mechanism and develop new treatments for ALD.

## Supplementary Material

Supplementary tables.

## Figures and Tables

**Table 1:** The top ten signal pathways were obtained according to Gene Ratio in QGHXR and ALD

	ID	Signaling pathway	Gene Ratio
QGHXR	hsa04080	Neuroactive ligand-receptor interaction	109/522
	hsa05022	Pathways of neurodegeneration - multiple diseases	79/522
	hsa05010	Alzheimer's disease	72/522
	hsa05417	Lipid and atherosclerosis	57/522
	hsa05163	Human cytomegalovirus infection	55/522
	hsa04020	Calcium signaling pathway	52/522
	hsa05012	Parkinson's disease	52/522
	hsa05169	Epstein-Barr virus infection	51/522
	hsa05020	Prion diseases	51/522
	hsa04151	PI3K-Akt signaling pathway	51/522
ALD	hsa04080	Neuroactive ligand-receptor interaction	56/271
	hsa04932	Non-alcoholic fatty liver disease (NAFLD)	47/271
	hsa05160	Hepatitis C	47/271
	hsa05417	Lipid and atherosclerosis	47/271
	hsa05164	Influenza A	42/271
	hsa05161	Hepatitis B	40/271
	hsa05162	Measles	39/271
	hsa05010	Alzheimer's disease	39/271
	hsa05171	Coronavirus disease - COVID-19	37/271
	hsa04151	PI3K-Akt signaling pathway	36/271

## Abbreviations

ALD: alcoholic liver disease
ALT: alanine aminotransferase
AST: aspartate aminotransferase
BP: biological process
CC: cellular component
EMT: epithelial-mesenchymal transition
ERS: endoplasmic reticulum stress
FDR: false discovery rate
GO: Gene Ontology
KEGG: Kyoto Gene and Genome Encyclopedia
MF: molecular function
PPI: protein-protein interaction
QGHXR: qinggan huoxue recipe
TC: total cholesterol
TCM: traditional Chinese medicine
TCMNPAS: TCM Network Pharmacology Analysis System
TG: triglycerides
